# Proposal of an Alpine Skiing Kinematic Analysis with the Aid of Miniaturized Monitoring Sensors, a Pilot Study

**DOI:** 10.3390/s22114286

**Published:** 2022-06-04

**Authors:** Caterina Russo, Elena Puppo, Stefania Roati, Aurelio Somà

**Affiliations:** Department of Mechanical and Aerospace Engineering, Politecnico di Torino, Corso Duca degli Abruzzi 24, 10129 Torino, Italy; elena.puppo@gabel.it (E.P.); stefania.roati@gabel.it (S.R.); aurelio.soma@polito.it (A.S.)

**Keywords:** monitoring system, alpine skiing, micro-electro-mechanical system sensors, sport engineering, training

## Abstract

The recent growth and spread of smart sensor technologies make these connected devices suitable for diagnostic and monitoring in different fields. In particular, these sensors are useful in diagnostics for control of diseases or during rehabilitation. They are also extensively used in the monitoring field, both by non-expert and expert users, to monitor health status and progress during a sports activity. For athletes, these devices could be used to control and enhance their performance. This development has led to the realization of miniaturized sensors that are wearable during different sporting activities without interfering with the movements of the athlete. The use of these sensors, during training or racing, opens new frontiers for the understanding of motions and causes of injuries. This pilot study introduced a motion analysis system to monitor Alpine ski activities during training sessions. Through five inertial measurement units (IMUs), placed on five points of the athletes, it is possible to compute the angle of each joint and evaluate the ski run. Comparing the IMU data, firstly, with a video and then proposing them to an expert coach, it is possible to observe from the data the same mistakes visible in the camera. The aim of this work is to find a tool to support ski coaches during training sessions. Since the evaluation of athletes is now mainly developed with the support of video, we evaluate the use of IMUs to support the evaluation of the coach with more precise data.

## 1. Introduction

The widespread use of wearable devices based on MEMS (micro-electro-mechanical system) sensors creates the possibility of sports monitoring even for non-professional users [1]. In the field of wearable technologies, one of the most popular is the inertial measurements unit (IMU). These devices let us measure a significant amount of information such as accelerations and angular velocity of a body applying only one sensor and allow us to compute the absolute and the relative angles of each application point [2,3]. For these reasons IMU are widely employed in different fields for diagnostic and monitoring purposes. With a miniaturized monitoring device, it is possible to reach a deep knowledge of the sports activity studied and to have the capability to understand the causes of injuries and adopt strategies to avoid them [4,5]. The data from the monitoring system provides the opportunity to track errors and enhance performance [6,7,8]. Error detection could help prevent and avoid injuries, help users in a rehabilitation program and help athletes improve their technique [9,10]. Especially in the field of sport and rehabilitation, these sensors are used to better understand the movements of athletes [11,12,13,14]. These sensors are also extensively studied in the literature combined with electromyography or video recording to ensure complete data in order to understand athletes’ movements and provide them with useful knowledge to improve their performance [15,16]. The number of articles on monitoring technologies to better understand athletes’ movements is constantly growing. Particularly noteworthy are the ones regarding the role of wearable technology in the understanding of the kinematics of a sport activity [17], not only in alpine skiing but also in other sport activities such as Nordic Walking; on that topic, see [18] and on cross-country skiing, see [19].

With regards to alpine skiing, it is very important to monitor and evaluate various parameters such as orientation, speed and also the possibility of recognizing the skier’s level [20,21,22,23,24]. IMUs are often used in combination with other sensors to ensure a better understanding of movements but above all to correct drift with sensor fusion algorithms. In alpine skiing, normally, the sensors used in conjunction with the IMU are the pressure or force sensors to monitor foot pressure during a ski run. The pressure on the plantar was used to correlate this data with the motion analysis obtained from the IMU; this information is used to tell an expert skier from a recreational one [25]. Also in the work of Bon et al. [26], pressure sensors in ski boots are used, combined with the Xsens MVN Link inertial suit system. Moreover, turn detection is a fundamental task for evaluating the difference between a carved turn and a drifted one [27,28]. In this research, information on the angular velocity and on the radial force has been added to discriminate between different skill levels of skiers. The GNSS is used as the golden standard for these measures and also for tracking trajectories, and the comparison of GNSS and AdMos (a GNSS receiver from Advanced Sport Instruments) is shown in the work of Jolstad et al. [29]. In the work of Fasel et al. [30], a method was adopted to correct drift in speed measurements by installing a reference magnet of the gate of a ski slope. This method allowed them to obtain the kinematics of the athlete’s center of gravity with greater accuracy and precision than a GNSS-based system (global navigation satellite system). This method is suitable for indoor monitoring without the use of GNSS. Another method used to compute the trajectory followed is unmanned aerial vehicle (UAV) and ground cameras supported by the neural network and the correlation filter-based algorithm [31]. In the work of Ruiz Garcia et al. [32], to avoid injuries and improve technique, the acceleration and inclinations of a skier during a turn are evaluated. The IMU angular evaluations are compared with the video reference analysis with optimum correlation results.

Focusing on the research that will be presented, the athletes were monitored with five IMUs. This set of sensors allowed us to evaluate the ski run, the number of curves, the ski boot cuffs, the poles and the lower trunk orientation. The principal aim of this work was to enhance the performance of athletes by supporting trainers with qualitative data.

## 2. Materials and Methods

### 2.1. Measurements

**Figure 1:**
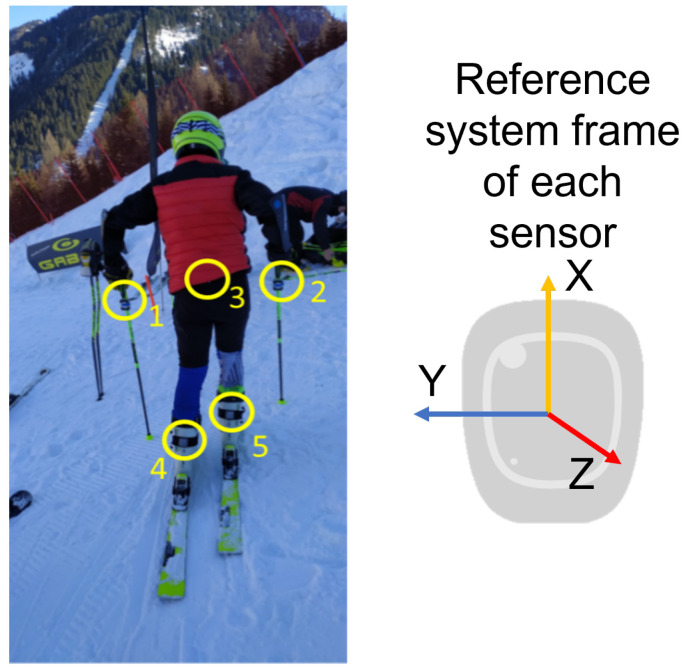
Sensors reference system.

The IMUs adopted for this work are five Mbientlab MetaMotionRs [33] that integrate three MEMS sensors: an accelerometer, a gyroscope and a barometer. The IMUs are firmly placed on the skier’s body, two of them are placed on the upper part of the rear of the ski boot cuffs, the other two on the poles, under the handle and on the lower trunk. We have chosen these sensor locations because we consider them the minimum number to monitor this complex activity. The ski boot sensors allow us to have the best focus concerning what happens at the level of the skis, the pole sensors allow us to understand the mobility of the upper body and the trunk sensor allows us to monitor the inclinations of the core. More sensors could be useful to obtain more information, for example, on the shoulders or knees, but we decided to install a reasonable number of sensors on the athlete so as not to interfere in his movements. The orientation is kept identical for each sensor to obtain a data set easier to analyze. The x axis points upward, the y axis points to the left and the z axis points back. Figure 1 shows the reference system of the IMUs and the reference of each sensor is collected in the following Table 1:

The technical specifications of the accelerometer and gyroscope are shown in Table 2 and Table 3:

The sampling frequency chosen was 50 Hz because this value allows noise removal and precise results [34]. The value was chosen after different tests both in the laboratory and in the field. We tested different sampling frequencies in order to find the best compromise between the accuracy of the results and the efficiency of the system. As regards the measuring range for the accelerometer, ±16 g and for the gyroscope ±2000°/s were used. For the resolution, the values were, respectively, 2048 counts/g and 16 counts/°. These parameters were identified to obtain a reliable dataset with a sustainable computational time during the post-processing. The data obtained from the IMUs were used to compute the angle of each body segment, relative to its reference frame, on which they were mounted. The barometer measures, sampled at 2 Hz, were used to compute the pressure decreases concerning the slope and these were correlated with the total height of the slope.

### 2.2. Data Analysis

**Figure 2:**
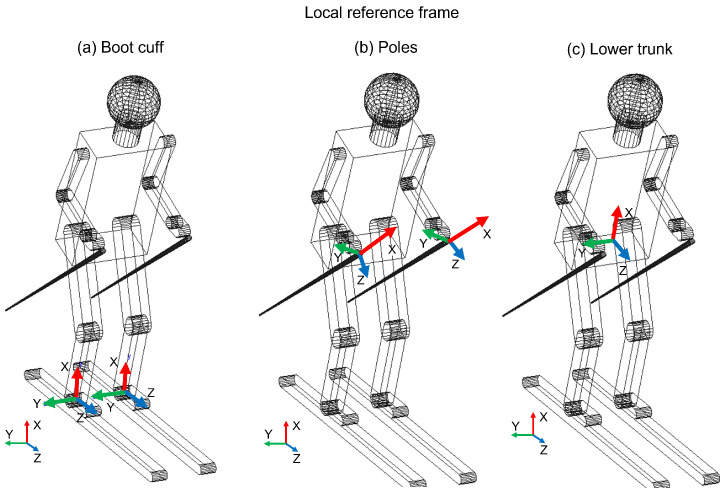
Local reference frame for each monitored part: boot cuff, lower trunk and poles.

The experimental activity was developed during ten Giant Slalom and Special Slalom runs by six agonist athletes (one female and five male) and a member of the World Cup team. The mean age of the participants was between 22 and 37. This activity took place in two different locations in Melette (IT) and Pozza di Fassa (IT) during the 2021 winter. The course setting was installed as a distance from the gates of between 18 and 20 m for the Giant Slalom and between 8 and 12 m for the Special Slalom; the lateral distance between the gates was 6–8 m and 2/3 m, respectively. For each sensor, it is possible to evaluate the characteristic angles (roll, pitch and yaw), explained in Table 4, and through these characterize the alpine skiing discipline. In Figure 2, the local reference frame for the boot cuff, the lower trunk and the poles is shown. The x axis is in red, the y axis is in green and the z axis is in blue. In the left-lower corner, the absolute reference frame is shown. The x axis has a leaning angle (16–18°) with respect to the vertical axis, due to the position of the sensors.

**Figure 3:**
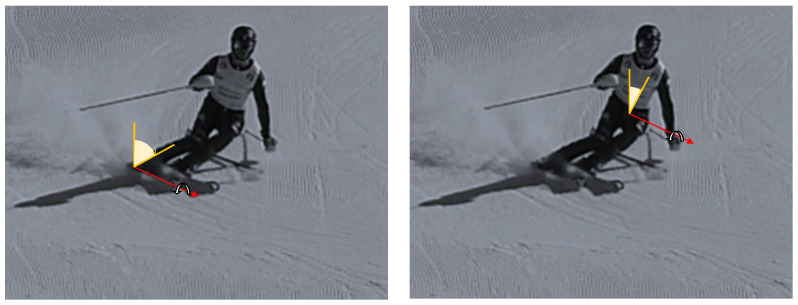
Lateral inclination for boot cuff and lower trunk.

**Figure 4:**
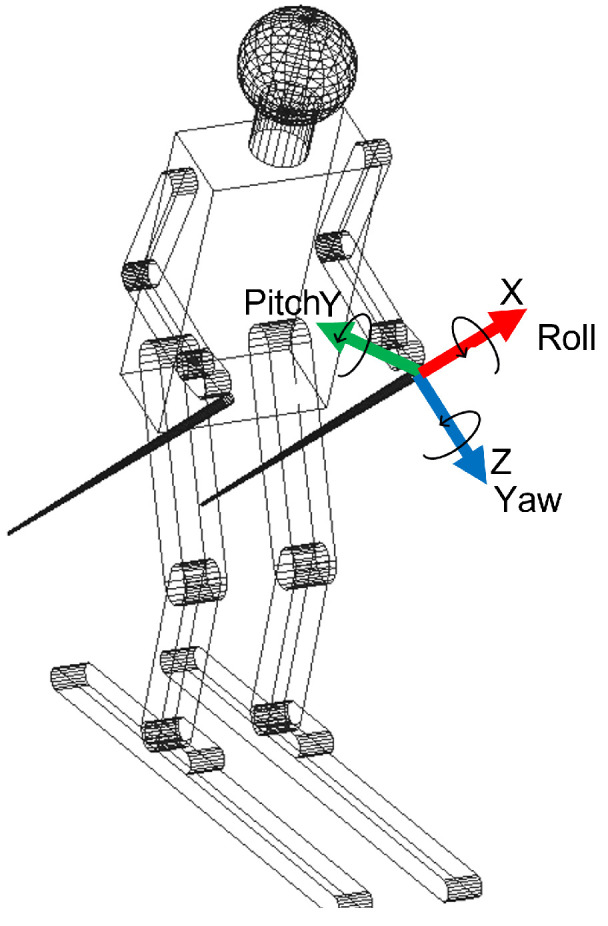
Pole roll, yaw and pitch angles.

1. The lateral inclination of the boot cuff and the lower trunk, shown in Figure 3, was evaluated with the roll. This represents the typical movement of the skier and can also be considered as an index of the athlete’s abilities. The rotation of the pole around the z axis represents the rotation of the pole in the traversal plane, as is visible in Figure 4.
2. The yaw angle for boot cuff and lower trunk represents the direction of the ski boot and skies during the ski run and it is directly connected with the trajectory performed. The rotation of the pole around the x axis is expressed by the yaw.
3. With the pitch angle, we measure an angle of a moving part of the ski boot with different stiffness regarding discipline and type of boots. The lower trunk indicates inversion or eversion. In [35], pitch angles varying up to 10° were experimentally observed. This variation changes based on the type of ski boots and on the level of the skier. For agonist skiers, such as the ones tested during the work presented in this paper, the pitch angle variation is reduced. So, in this work, due to the high level of the skier and to the comparison developed, this parameter was neglected. This consideration is not applicable for the poles because their local reference frame is decoupled from the slope and the pitch can be evaluated.

**Figure 5:**
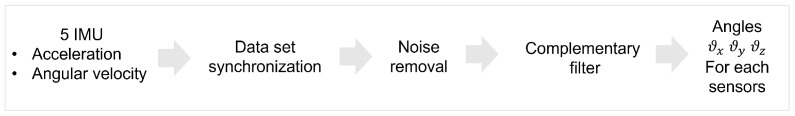
Followed algorithm for the data analysis.

In this section, the algorithm followed to analyzed the data will be presented. The path followed is summarized in the image below (Figure 5):

With the complementary filter, the roll and the yaw angles were obtained combining the information of the accelerometer and the gyroscope. Due to the speed during the turns, the large size of the angle $\theta_{tot}$ was given by the angular velocity that describes the turns correctly. The information of the accelerometer was used mainly to correct the drifting of the gyroscope. First, the accelerometer signal was filtered with a low-pass filter with a cut-off frequency of 5 Hz; then, the trigonometric formulas were used to compute the orientation values of the body during the ski run. The angle $\theta_{acc}$ is evaluated for each axis, measuring the static inclination (Equation (1)). Due to the dynamics of the sport monitored, the angles evaluated with the accelerometer are used only to correct the drift of the angle computed with the gyroscope. In fact, preferably, MEMS (micro-electrical-mechanical system) could be used to measure the static orientation of the sensors, in particular the orientation of an axis with respect to gravity.

(1) $$\theta_{x,acc}=\arctan\frac{a_{x}}{\sqrt{\left(a_{y}^{2}+a_{z}^{2}\right)}}$$

(2) $$\theta_{y,acc}=\arctan\frac{a_{y}}{\sqrt{\left(a_{x}^{2}+a_{z}^{2}\right)}}$$

(3) $$\theta_{z,acc}=\arctan\frac{a_{z}}{\sqrt{\left(a_{y}^{2}+a_{x}^{2}\right)}}$$

The angular velocity, on the other hand, is filtered by a high-pass filter with a cut-off frequency of 0.1 Hz and it is then integrated to calculate the angle.

(4) $$\theta_{gyro}=\int \omega dt$$

So to compute the total angle for each axis, the complementary filter is used:

(5) $$\theta_{tot}=\alpha \cdot \theta_{acc}+\beta \cdot \theta_{gyro}$$

The $\theta_{tot}$ computed with this algorithm is the orientation of each body segment monitored during the ski run. The parameters α and β are the coefficients of the filter used to indicate how relevant the angle computed with the gyroscope is with respect to the accelerometer. In this case, due to the high dynamics and velocity of the rotation, the gyroscope measures the orientation of the sensor better, and the orientation computed with the accelerometer is used only to correct the drift. The algorithm used was tested with a calibration phase where the parameters α and β were set to the following values, in accordance with the values in the literature [36,37]: the alpha was equal to 5% and beta to 95%. With these angles, it is possible to obtain different information regarding the run performed, which, combined with the expert assessment of a ski coach, can provide useful corrections for the athlete’s technique. This allows the ski coach to obtain a more complete assessment of the skier during the training phase.

**Figure 6:**
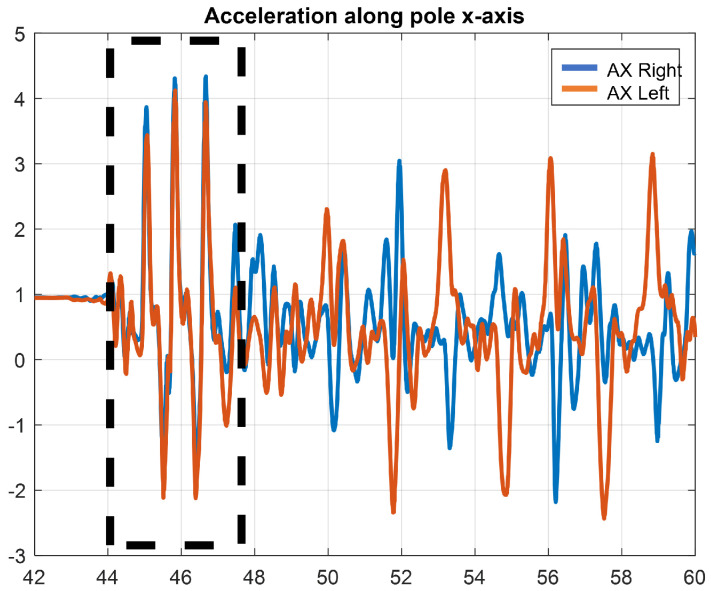
Pole acceleration along x axis.

The MetaBase application developed by the company Mbientlab [33] was used during the data collection session. Through this application, it is possible to start the data collection of the five sensors at the same time. In order to have a unique starting frame, the accelerations of the poles was used. In particular, in the accelerations along the x axis, the pushing phase during which the athletes pushed themselves off to start the ski run is clearly visible. The three initial peaks in Figure 6 represent the pole pushing.

Then the time of the first peak is taken as the starting point for each run, and all the data collected are cut starting from this reference time.

## 3. Results

From the post-processed data, it was possible to obtain a large amount of data regarding the ski run, such as the turn definition (number of turns and the average time of each turn) and the average roll and yaw angle for each node monitored (boot cuff, lower trunk and poles).

**Figure 7:**
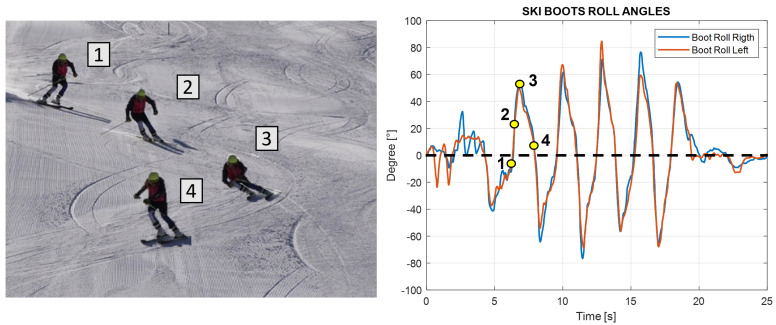
Highlight of one turn in the roll angle graph.

**Figure 8:**
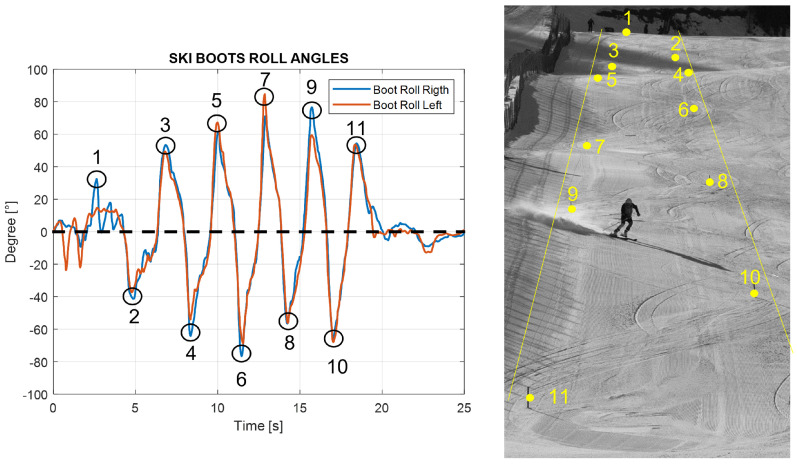
Number of turns in video and roll angle.

- **Turn definition**: The beginning and the end of the turn can be defined starting from the edge change visible in the boot roll angle graph. The peaks in this graph represent the maximum lateral inclination of the ski boot during a turn; this occurs in the central phase of the turn when the roll curve change sign corresponds to the edge change and corresponds to the start of the consecutive turn. Hence, each turn can be calculated using zero as a reference, as shown in Figure 7 below.
- **Average time of turns**: For each turn, it is possible to compute the time from the start of the turn and its end and compute the average for all of them. This time can be computed considering the starting point when the boot cuff yaw angle is at a maximum, which indicates the edge change as the start of a turn. This yaw angle theoretically should coincide with the zero of the roll angle of the boot cuff. The turns are easily visible in the roll angle graph. The angle value oscillates around the zero mean value; the peaks instead represent the maximum inclination of the skier during the turn. Around the zero value, the ski boot has no inclination and the skier is between two turns; at the peak values, the skier is in the middle of the turn. To know the finish time, the number of peaks in the roll angles plot is counted and compared with the number of gates to validate it.
- **Number of turns**: Counting the peaks from the roll angle graph, the number of turns performed is obtained. The procedure is shown in Figure 8, where on the left the counting peaks and on the right the same turns shown in the ski slope are visible.
- **The average roll angle for ski boots and for the back**: Computing the maximum inclination of the roll angle values, the average lateral inclinations of the skiers during each turn is obtained.

**Figure 9:**
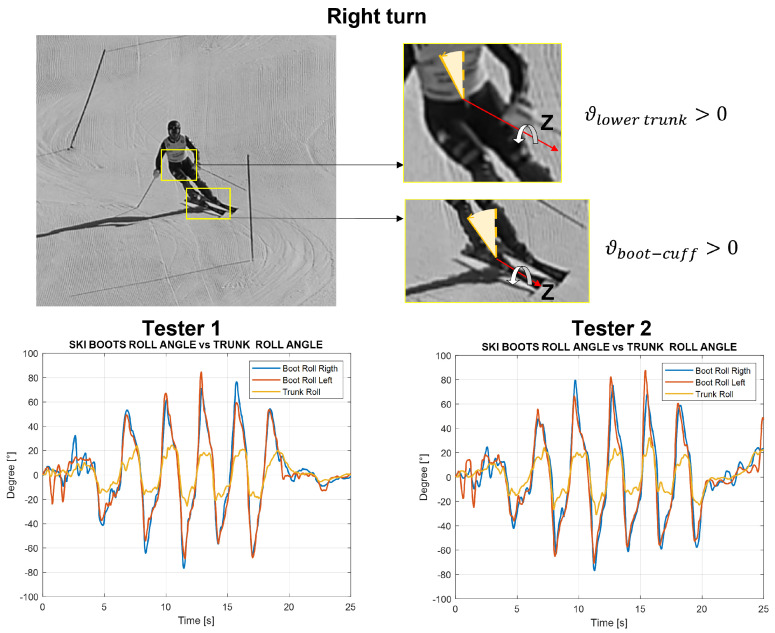
Roll angle for the ski boots and for the back.

**Figure 10:**
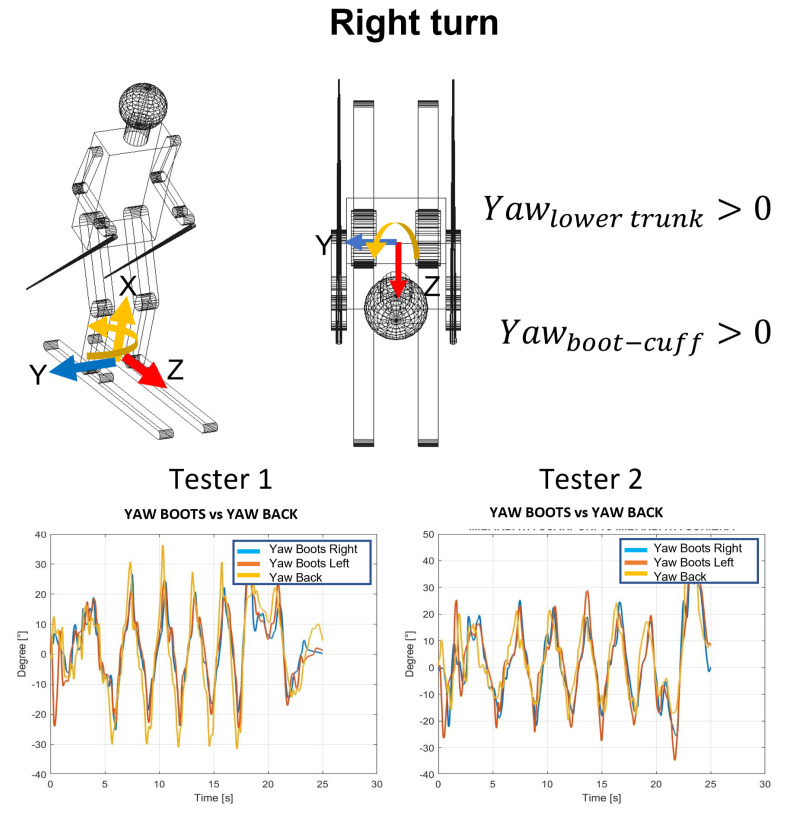
Yaw angle for the ski boots and for the back.

**Figure 11:**
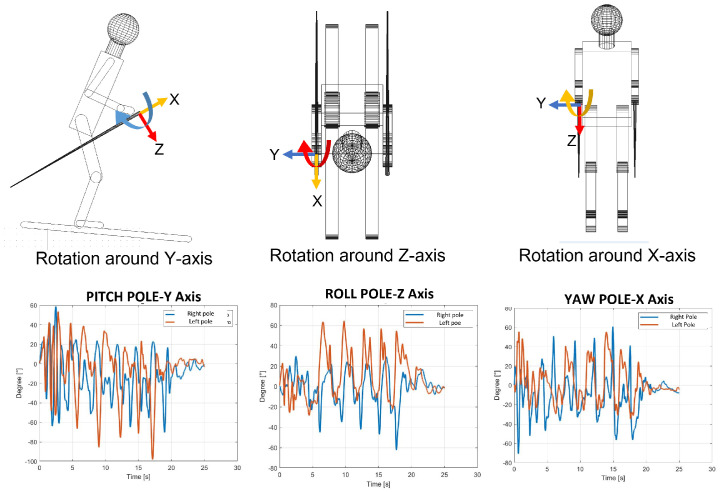
Roll, yaw and pitch angles for poles.

From Figure 9, it can be seen how the mean peak value of the back roll angle always remains lower with respect to the ski boots. This is because the trunk has to remain almost perpendicular to the slope to keep balance and balance the forces during turns. In Table 5, the average values for the roll peak angles for ski boots and back are shown:

- **The average yaw peak angle for ski boots and for the back**: The yaw angle values oscillate around zero. The zero position represents the ski orientation alongside the slope; the maximum inclination represents the ski oriented with this angle with respect to the slope. So, in Figure 10, the peaks represent the end of each turn, and the zeros represent the central phase of the turn. In Table 6, the average values of yaw angles are reported.
- **The average angles for poles**: The poles’ orientation became an interesting parameter to understand the correct posture during the ski run. From the angles computed, it is possible to observe the three different movements of the poles. In Figure 11, these movements are shown with the three rotations of the poles. The roll angle of the poles should remain very similar to the lower trunk roll angle because it shows athletes that perform with their arms and poles close to their body, keeping a correct posture. The yaw angle highlights the movement of the poles following the direction of the skies. At the end, the pitch angle emphasizes the understanding of the tendency to approach the gate with arms, which represents imbalanced behavior in the athletes.

### 3.1. Results Interpretation

**Figure 12:**
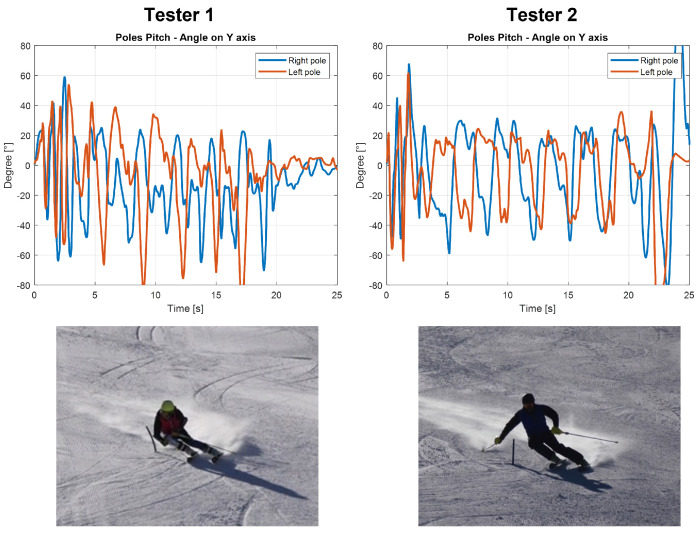
Pitch angle for right and left pole for Testers 1 and 2.

In this subsection, the main information extrapolated from these results is explained. The principal aim of a ski coach is to evaluate the ski run, identify the errors and propose a strategy to fix them to the athletes. Due to the high level of ski preparation of the athlete’s tested, evident mistakes were not found during this work. At this level, the errors are subtle and should be called inaccuracies. These regard mostly the inclination toward the inner part of the turn and the balancing in the rotation between the trunk and the lower body. For less expert skiers, these inaccuracies became more evident, in particular regarding the position of the body that must lean forward and the central position on the skies. These errors are easily identified by the coach; the actual procedure for the evaluation of the ski run during training is just the comments after a run or the video recording supported by comments from the coach. With the IMU measures, the evaluation could be more complete both for the coach and for the athletes. Therefore, it is mandatory to develop another training session with less-experienced skiers to identify more clearly the differences. Regardless, from the data collected it is possible to identify some behavioral characteristics that are common even among high-level athletes, for example, excessive movements of the upper body, but also some information regarding each turn in order to understand the weaknesses of the ski run and how the athlete has to improve. With the help of these data, the suggestion that the ski coach can offer could be more specific and precise for each athlete. In the case of a less expert skier, for example, if he misses a gate the coach could immediately see the error from the data without seeing the video because there is a missing part in the graph of the roll or yaw of the ski boots. For a more advanced skier, it is possible to evaluate the lateral inclination of each ski boot and the correct use of poles during the ski run. With the data collected on the poles, the possibility of evaluating the amplitude of movements of athletes’ arms was observed. The skiing technique requires a very balanced posture, and a strong movement of the upper body and arms can generate an unbalance and consequently a reduction of speed or the incorrect entering in a turn. From the video, it was observed that Tester 1 has evident arm and pole movements during the ski run; in fact, as can be seen in Figure 12, the athlete opens and closes his arms approaching the turn. On the other hand, Tester 2 keeps his arm more still during the entire run. The same can be said from the data collected through the pitch angles; in fact, a more stable value of the peak values of Tester 2 was obtained. In contrast, the graph of the pitch angle for Tester 1 is more segmented and presents more spikes.

Despite this imbalance in the upper body movements, Tester 1 performs the ski run more efficiently (17.87 s of total time vs 18.29 s for Tester 2), finishing the slope faster. However, in order to improve his technique, it was mandatory that his coach pointed out this behavior in order to fix it. In fact, it would be more useful if the posture that he kept during the turn were maintained during all the slopes to complete a more efficient run. These considerations are fundamental and preparatory to the identification of evaluation indexes. These indexes are useful to evaluate the ski run of each athlete and compare different runs. The speed, the time between two gates and the lateral inclination of the ski boots are usable information in the definition of these indexes. A new test session is mandatory to pursue this purpose. Only in this way is it possible to evaluate whether these indices evaluate the different abilities among athletes.

### 3.2. Comparison of Video and IMU Data

**Figure 13:**
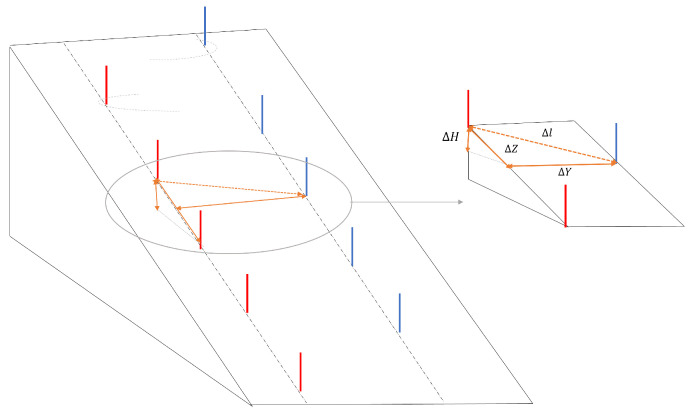
Slope inclination measures.

First of all, the IMU data were synchronized using the first pushing phase of each athlete at the starting phase. The pushing phase is clearly visible from the data obtained from the accelerometer placed on the poles, especially along the x axis that is aligned with the longitudinal axis. With the software, Kinovea, the recorded video of the test was analyzed. Each video was synchronized temporally with the data measured with the IMUs using the chronometer tool available in the software and made it start during the first push on the poles. Then, in order to obtain a comparison between the angles measured by the sensors and the ones recorded from the video, a projection of the IMU ones is mandatory. The angles computed with the IMU are in a local reference frame; in contrast, the angles recorded with the camera are in a global reference frame. To compare them, evaluation of the slope is necessary. To obtain the slope, barometric measures were used in combination with the information about the ski setting. In order to compute the slope of each segment of the ski run, we assumed the distance between two gates was about 9 m (ΔZ in Figure 13) and the lateral distance (ΔY) was about 10 m and we measured the height with the barometer (ΔH). This approximation allows us to obtain the rotation matrix for each curve to compare the video data with the IMU ones. The rotation matrices were used to multiply the roll and yaw angle in order to compute the same angles in the global reference frame. The use of the barometer allows a first approximation of these matrices that, in future work, have to be computed with a more accurate method such as GNSS.

**Figure 14:**
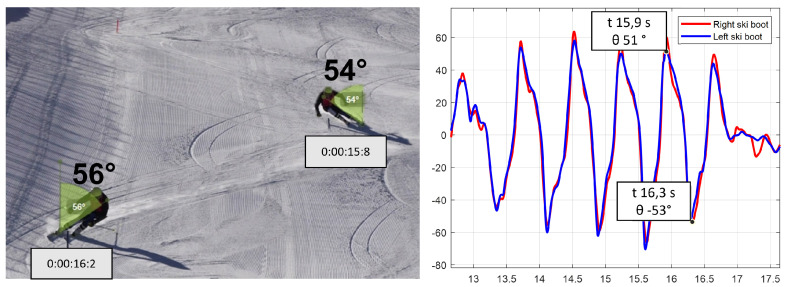
Comparing the video roll angles with the roll angles computed with the IMU.

Once the slopes of each part of the ski run are obtained, it is possible to project the roll and the yaw measured with the IMU in the absolute reference. A good comparison was obtained, giving confidence in the algorithm used. In future work, more accurate validation [38] with 3D motion capture simulation has to be performed in order to be able to also validate the boot cuff angles in the other planes and also the lower trunk and pole angles and to compute the inclination of the slope properly. We show the result obtained for two consecutive turns of a ski run:

Tester 1 was Giant Slalom (see Figure 14).

From the analyzed data, an inclination of 54° at a time of 15.8 s was identified for the left boot during the curve from left to right, which has the left ski as external. An inclination of 56° at the time of 16.2 s was identified in the next turn, with the right ski as external. The results obtained show agreement between the video angles and the IMUs. To also compare the yaw angle and the pole angle, tridimensional information from the video is necessary, which can be obtained only with a deeper video analysis. Performing this comparison enables a better understanding of the values computed with the IMUs that are reasonable in the alpine skiing contest. As mentioned before, a 3D motion-capture video analysis has to be performed in order to obtain more precise data regarding all the three-axis angles, but this comparison offers an index of the right path we are following. In this comparison, the percentage quote of human error, due to the positioning of the sensors and due to the manual insertion of the start timing in the video recording and the inclination of the camera, must be inserted. Moreover, the technical error intrinsic in the measure of an IMU must also be inserted.

## 4. Discussion

The main aim of this work was proposing a wearable IMU-based monitoring system for the evaluation of the ski run. A monitoring structure, based on different application points, was realized to improve athlete performances, and provide the coach qualitative data to support his evaluation. The monitoring device is meant to be worn during the training sessions and the data collected and the coach’s evaluation of these data allow technique improvement. The monitoring devices based on wearable sensors are easily and intuitively worn during a ski run. The number of application points (upper limbs, lower limbs and trunk) gives a complete evaluation of the gesture and the kinematics of each joint. The turn detection in this work is defined using ski boots’ roll angles. When the roll angle curve changes sign, this identifies the edge change of the ski and the start of a new turn. In this pilot work, the focus of the work was to develop and find some indication from the sensors that could be used by the trainer to improve his evaluation of an athlete. As Martinez et al. [24,27,28] and Snyder et al. [23] mentioned in their work, the identification of the turn with the roll angle is valid if the parallel turns are performed and if the athlete is an expert. They developed a sophisticated algorithm to identify the turn for different types of turns (parallels, drifted and snowplow) and at the same time to identify the levels of different skiers. Video recordings were used in this work to compare the IMU angles with the video ones but also to start from the coach’s standard way of evaluating an athlete. We consider the evaluation of the trajectory not fundamental for the proposed work. The use of GNSS and other methods of localization presented by Jølstad et al. [29] and Fasel et al. [30] is useful if we exclude the video recording. Moreover, the use of pressure sensors, as explained in the work of Bon et al. [26] and Matsumura et al. [25], we consider to provide useful information but not to be fundamental for this pilot work in which we preferred the easy application of five sensors for the kinematics data. With the data acquired, the information that can be extracted from them was shown. Especially with the main angles, which characterize the alpine skiing discipline, it is possible to obtain a variety of information regarding the quality of the ski run. In fact, it is possible to extract some interesting information by analyzing each turn separately and synchronizing the different sensors. This paper showed the roll angle evaluation of the boot cuff and its comparison with the data obtained from the video frames, obtaining a promising agreement concerning these data. The data coming from the poles can also add some interesting information about the arms’ and upper limbs’ movements during a ski run. This information can be useful to understand the right balance between the upper and the lower part of the body to guarantee an optimal ski run. The limits of this work are mainly due to the reduced number of skiers tested, both in terms of numbers and in terms of ski levels. It would be useful to test a larger group of testers with a high variety of ski levels to be able to distinguish an expert skier from a lower-lever skier. However, a more detailed 3D motion capture validation would be required to complete this work. Introducing, in future work, a comparison between measured data and a simulation of an anthropomorphous multibody model would help in the evaluation of the characteristic angles explained in this work. We also want to consider the possibility of evaluating a skier’s experience based on the ski boot roll angles. The higher the roll angles, the higher the expertise level of the skier will be, but we need more data to prove this consideration.

## 5. Conclusions

In conclusion, the aim of this work is to provide the ski coach and the athlete with a larger amount of data on which to base the evaluation of a ski run. Up until now, the evaluation of a ski run was developed only using video and the information given by a coach; with this monitoring tool, the video information can be correlated with some graphs that can be useful for technique improvement. In particular, it can be useful for observing the unbalance between the upper body and the lower body, for observing whether the athlete reaches the gate with an arm, for seeing if it is necessary to improve the lateral inclination of the skies during a turn and for evaluating the correct timing of a weight movement approaching a turn.

## Figures and Tables

**Table 1:** Reference number of each sensor.

Number	Name
1	Left pole
2	Right pole
3	Lower trunk
4	Left boot cuff
5	Right boot cuff

**Table 2:** Accelerometer technical specification.

Description	Min	Max	Units
Measurement range	±2	±16	g
Resolution	2048	16,384	counts/g

**Table 3:** Gyroscope technical specification.

Description	Min	Max	Units
Measurement range	±125	±2000	°/s
Resolution	16	262	counts/°

**Table 4:** Description of the characteristic rotations around the sensor axis.

Angles	Description
Roll or Lateral Inclination	Rotation around the z axis
Yaw or turn	Around the x axis
Pitch or flex	Rotation around the y axis

**Table 5:** Average roll angles for ski boots and back.

Tester	Av. Ski Boot Roll Peak Angles	Av. Back Roll Peak Angles
1	62°	23°
2	63°	21°

**Table 6:** Average yaw angles for ski boots and back.

Tester	Av. Ski Boot Yaw Angles	Av. Back Yaw Angles
1	22°	29°
2	21°	29°

## Data Availability

Not applicable.
